# Indiana State Medical Society

**Published:** 1868-04-15

**Authors:** L. D. Waterman

**Affiliations:** Sec. Ind. St. Med. Soc.; Indianapolis


***Indiana State Medical Society.***

INDIANAPOLIS, March 29, 1868.

*Editor Chicago Medical Journal :*

Please mention in your April number that the Indiana State Medical Society will meet at Indianapolis, on Tuesday, May 19th, 1868.

Very respectfully,

L. D. WATERMAN,

*Sec. Ind. St. Med. Soc.*

